# Three Dimensional Vero Cell-Platform for Rapid and Sensitive Screening of Shiga-Toxin Producing *Escherichia coli*

**DOI:** 10.3389/fmicb.2019.00949

**Published:** 2019-05-07

**Authors:** Celina Z. To, Arun K. Bhunia

**Affiliations:** ^1^Molecular Food Microbiology Laboratory, Department of Food Science, Purdue University, West Lafayette, IN, United States; ^2^Department of Comparative Pathobiology, Purdue University, West Lafayette, IN, United States; ^3^Purdue Institute of Inflammation, Immunology and Infectious Disease, Purdue University, West Lafayette, IN, United States

**Keywords:** Shiga-toxin producing *Escherichia coli* (STEC), cytotoxicity, Vero cells, 3D, food ground beef, multiplex-PCR, pathogen detection

## Abstract

Shiga-toxin producing *Escherichia coli* (STEC) is a serious public health concern. Current Vero cell assay, although sensitive, is lengthy and requires 48–72 h to assess STEC presence in a sample. In this study, we investigated if Vero cells in a three-dimensional (3D) platform would provide improved sensitivity for rapid screening of STEC. Vero cells (epithelial kidney cell line) were grown as a monolayer (2D) or in a collagen-matrix (3D) and exposed to Shiga-toxin (Stx) preparation or STEC cells that were pre-exposed to antibiotics (mitomycin C, ciprofloxacin, or polymyxin B) for toxin induction. Lactate dehydrogenase (LDH) release from Vero cells was used as a biomarker for cytotoxicity. Modified tryptic soy broth (mTSB) as enrichment broth containing mitomycin C (2 μg/ml) or ciprofloxacin (100 ng/ml) significantly induced Stx production, which was further confirmed by the dot-immunoblot assay. The 3D Vero platform detected STEC after 6 h post-infection with cytotoxicity values ranging from 33 to 79%, which is considerably faster than the traditional 2D platform, when tested with STEC. The cytotoxicity for non-Stx producing bacteria, *Salmonella*, *Listeria*, *Citrobacter*, *Serratia*, and *Hafnia* was found to be below the cytotoxicity cutoff value of 15%. The detection limit for the 3D Vero cell assay was estimated to be 10^7^ CFU/ml for bacteria and about 32 ng/ml for Stx in 6 h. STEC-inoculated ground beef samples (*n* = 27) resulted in 38–46% cytotoxicity, and the bacterial isolates (*n* = 42) from ground beef samples were further confirmed to be *stx1* and *stx2* positive in a multiplex PCR yielding a very low false-positive result. This 3D cell-based screening assay relies on mammalian cell pathogen interaction that can complement other molecular techniques for the detection of cell-free Stx or STEC cells from food samples for early detection and prevention.

## Introduction

Shiga-toxin (Stx) producing *Escherichia coli* (STEC) is of major public health concern and is one of the top five foodborne pathogens responsible for a high number of hospitalizations in the United States each year (Scallan et al., 2011). STEC comprises more than 200 serotypes and is Gram-negative, rod-shaped, non-spore-forming bacteria that live in the intestinal tract of animals, contaminated soil and surface waters (Mathusa et al., 2010). However, most do not cause serious illness unless it carries the Locus of Enterocyte Effacement (LEE) Pathogenicity Island that contains *eae* and genes for the Type III secretion system (T3SS) (Bhunia, 2018). Under severe cases, the infection can progress and lead to hemolytic uremic syndrome (HUS). Although some LEE-negative STEC strains can still cause illness, all outbreak strains that are highly associated to HUS are predominantly LEE positive strains (Hughes et al., 2006). The major serotypes of concern are O157, O26, O45, O103, O111, O121, and O145, which were responsible for several foodborne outbreaks (Martineau et al., 2001; Grant et al., 2011; Farrokh et al., 2013). The O157 STEC can be distinguished from other serovars based on their ability to ferment sorbitol. Sorbitol-positive species can either be O157:NM, non-O157 STEC, or non-STEC, and the sorbitol-negative species are O157 STEC (CDC, 2006; Pollock et al., 2010; Parsons et al., 2016). STEC can produce two types of Stx, Stx1, and Stx2, which are further subdivided into, Stx1a, Stx1c, Stx1d, Stx2a, Stx2b, Stx2c, Stx2d, Stx2f, and Stx2g, where Stx2a and Stx2c are the most prevalent subtypes that have been associated with HUS in patients (Sheoran et al., 2003; Bhunia, 2018). Therefore, advanced technologies and methods should be exploited for rapid detection of STEC including emerging pathogens that express *stx* gene to reduce the risk of food contamination, prevent foodborne outbreaks, and alleviate financial burden in the food industry.

Although mortality is low, the consumption of food contaminated with STEC leads to high morbidity (Karmali et al., 2010; CDC, 2012; Sperandio and Pacheco, 2012). Continuous efforts are being made to develop microbial pathogen and toxin detection platforms for improving food safety and diagnostic testing (Tokarskyy and Marshall, 2008; Wang et al., 2012; Bhunia, 2014; Cho et al., 2014; Tang et al., 2014; Wang and Salazar, 2015). According to the FDA and USDA-FSIS, a zero-tolerance policy is enforced in the United States where raw commodity must be free of the seven serogroups (O26, O103, O45, O111, O121, O145, and O157:H7) before retail distribution (Babsa et al., 2015;FSIS, 2016; Brusa et al., 2017).

Traditional culturing methods, although accurate, are tedious and lengthy. Further, the standardized methodology is only established for O157 serotype of STEC, limiting the ability to detect and quantify non-O157 STEC serotypes (FDA, 2001). Biochemical and physiological characteristics can be used to differentiate STEC O157 from non-pathogenic *E. coli*; however, such methods may not be able to distinguish non-O157 STEC from non-pathogenic *E. coli* (FDA, 2001). Molecular assay tools such as PCR and immunoassays are widely used (Tate and Ward, 2004; Medina et al., 2012; Schrader et al., 2012; Wang et al., 2016; Armstrong et al., 2018) but they may fail to differentiate viable from dead cells or active from inactive toxins. Moreover, these methods may have limited specificity due to the cross-reactivity of antibodies and performance may be hampered by inhibitors from complex food matrices. Mammalian cell-based assays have a unique advantage over other molecular techniques because of its ability to measure the physiological function and toxicity of the analyte (Banerjee and Bhunia, 2009; Banerjee et al., 2010). Furthermore, they may be suitable for rapid high throughput screening to rule out negative samples while positive samples can be examined by a confirmatory test, to achieve results in an hour to a day (Banerjee and Bhunia, 2009; Bhunia, 2014). To improve stability, sensitivity, and physiological relevance of the cell-based assays, mammalian cells are generally embedded in the natural matrices such as collagen, gelatin, elastin, silk fibroin, chitosan, chitin, fibrin, fibrinogen or grown as spheroid as a three dimensional (3D) scaffold, which mimics *in vivo* tissue organization and behavior and is an attractive model to study pathogen, toxin and drug interactions (Edmondson et al., 2014; Ravi et al., 2015; Barrila et al., 2018).

The Vero cell assay is considered the gold standard for screening Stx-positive samples in 48–72 h by examining the morphology of Vero cells under an inverted microscope (Konowalchuk et al., 1977). The assay was modified by our group to rapidly detect and differentiate STEC from non-pathogenic *E. coli* relying on the release of lactate dehydrogenase (LDH) from Vero cells as a biomarker for cytotoxicity and detection was achieved in 12–16 h (Roberts et al., 2001). Maldonado et al. (2005) used the same assay to evaluate the cytotoxicity profile of *E. coli* isolates from food and environmental sources. Later, Quiñones et al. (2009) modified the assay using a genetically modified Vero cell line with a destabilized variant (half-life, 2 h) of the enhanced green fluorescent protein (Vero-d2GFP) that could detect 100 pg/ml of Stx2 in 16 h. Infectious dose for Stx2a is reported to be 1–2,000 ng and for Stx1a is about 400 ng (Tesh et al., 1993; Russo et al., 2014). Although the assay has a low detection limit, the detection time is comparable to the previously mentioned LDH release assay (Roberts et al., 2001). Interestingly, commercial and newly developed cell-based assays only target Stx toxin, rather than STEC bacteria as the analyte. Furthermore, for best practice, a 6–8 h work shift is typically desired by both the food industry and government agencies. Thus our goal was to modify the Vero cell assay platform for improved sensitivity and rapid detection within the desired work-shift hours for STEC bacteria.

In this study, besides Vero cells, we also evaluated THP-1 (human monocyte cell line) for cytotoxicity, where both cell lines were embedded in a collagen matrix in a three dimensional (3D) assay platform and optimized assay conditions such as antibiotic induction for toxin production for testing with artificially contaminated ground beef samples. Samples were further confirmed for the presence of STEC (and Stx) by using the traditional culturing method and a multiplex PCR targeting *stx1* and *stx2*. Overall, results support the potential application of 3D Vero cell platform over the THP-1 platform for detection of STEC from food samples in 6 h after an initial enrichment step.

## Results

### Vero Cells Are More Sensitive to Purified Stx Than THP-1 Cells in a 3D Platform

**FIGURE 1:**
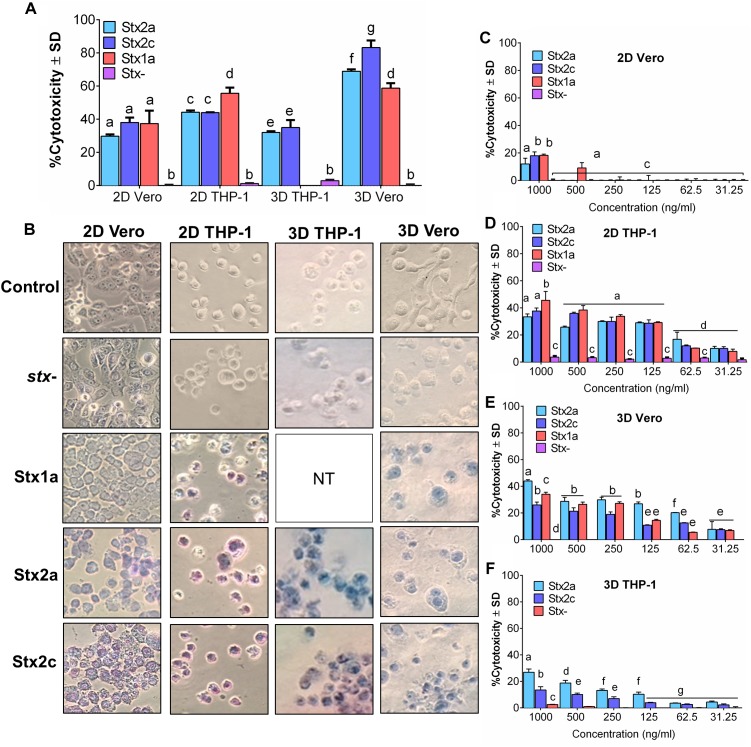
Vero cells are more sensitive to purified Stx than THP-1 cells in a 3D platform. Comparison of sensitivity against **(A)** commercial Stx1a, Stx2a, and Stx2c toxins at 1,000 ng/μl against 2D and 3D Vero and THP-1 cells after 16 h. **(B)** Visual examination of Vero and THP-1 cells using Trypan blue staining captured at 400× after 16 h post-infection. Cytotoxicity response to different concentrations (31.25–1,000 ng/μl) of commercial Stx1a, Stx2a, and Stx2c toxins to **(C)** 2D Vero, **(D)** 2D THP-1, **(E)** 3D Vero, and **(F)** 3D THP-1. Cell-free supernatant from *stx*^-^ strain (O157:H12 strain 489) was used as a negative control. Due to the limitation of resources, Stx1a was not tested for 3D THP-1 cells.

Initially, Vero cell monolayer (2D) and THP-1 cell suspensions in a 48-well tissue culture plate were tested for their sensitivity to purified Stx (1 μg/ml) after 16 h of exposure. In 2D setup, THP-1 cells showed significantly higher sensitivity to purified commercial Stx toxin subtypes than the Vero cells, and the corresponding Trypan blue stained microscopic cell images confirmed cell death characterized by cell rounding, detachment, and dye uptake (Figure 1A,B). Both cell lines were then embedded in collagen matrix using rat-tail collagen type I in a 48-well plate as before to create a 3D cell structure (Banerjee et al., 2008) and tested against purified Stx preparations. Data show that the 3D Vero cells were more sensitive to Stx than the 3D THP-1 cells (Figure 1A,B). In contrast, 2D THP-1 is more sensitive to Stx than the 2D Vero (Figure 1A,B). Next, we determined the limit of detection for each platform to purified toxins diluted serially and found that cytotoxicity response is concentration-dependent (Figure 1C–F). In both 2D Vero and 3D THP-1 assays, the detection limit was 0.5–1 μg/ml and cytotoxicity values varied from 20 to 30% after 16 h exposure. While the 3D Vero and 2D THP-1 cell platforms detected the Stx subtypes as low as 62.5–125 ng/ml. Collectively, these data indicate that the 3D Vero cell platform provides the best response to Stx thus was selected for further assay development.

### The Sensitivity of 3D Vero Cell Platform to STEC Cells

**FIGURE 2:**
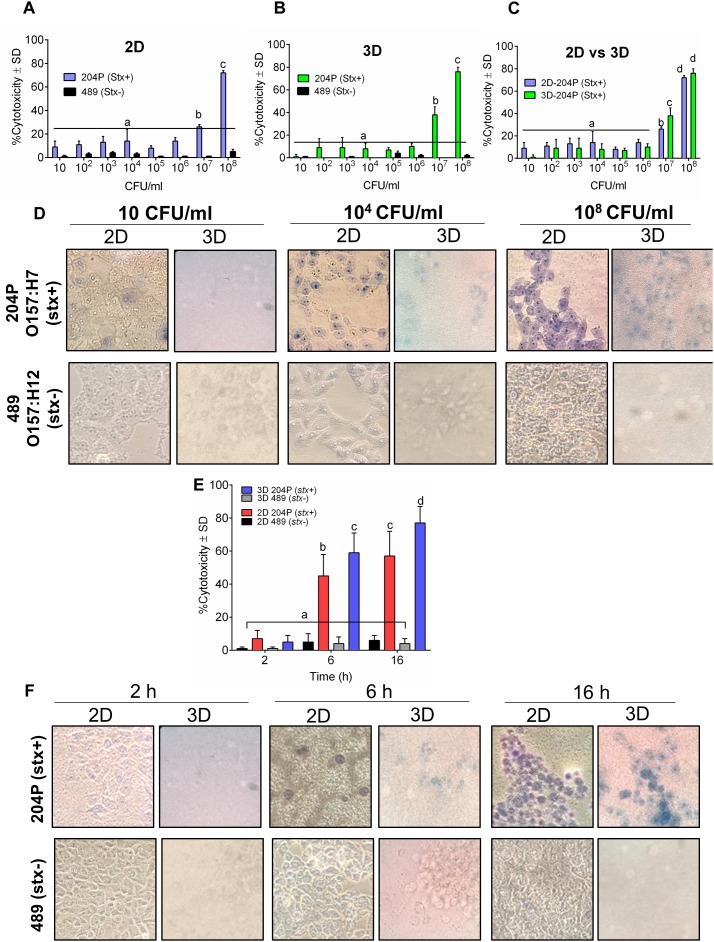
3D Vero cell platform is specific for STEC cells. Analysis of cytotoxicity of *stx*^+^ (O157:H7 strain 204P) and *stx*^-^ (O157:H12 strain 489) in **(A)** 2D Vero and **(B)** 3D Vero, and **(C)** 2D vs. 3D Vero cell assay. **(D)** Microscopic images showing the cytotoxicity effect of increasing concentrations of *stx*^+^ (204P) and *stx*^-^ (489) *E. coli* cells at 16 h under 400× magnification. **(E)** Cytotoxicity effect of *stx*^+^ (204P) and *stx*^-^ (489) *E. coli* cells (1 × 10^8^ CFU/ml) at 4, 6, and 16 h using 2D and 3D Vero cells and corresponding **(F)** light microscopic images under 400× magnification.

Next, we tested the sensitivity of 2D and 3D Vero cell-platforms after exposure to *stx*^+^ and *stx^-^* STEC cells after 16 h exposure at different cell concentrations. In both 2D and 3D platforms, a minimum of 10^7^ CFU/ml STEC was required to yield a positive response (Figure 2A–D and Table 1). The cytotoxic response from the 3D Vero platform (38 ± 7%, *P* < 0.05) was significantly higher (*P* < 0.05) than the 2D Vero platform (26 ± 2%) after exposure to 10^7^ CFU/ml of STEC (Figure 2A–C). Exposure to a higher concentration of STEC (10^8^ CFU/ml) induced significantly (*P* < 0.05) higher levels of cytotoxic response (72 ± 2 vs. 76 ± 4%) (Figure 2C). Cytotoxicity values of *stx^-^* strain (489) tested in either 2D or 3D platform (0–5 ± 1%) were significantly lower than the *stx*^+^ (204P) strain (*P* < 0.05). Microscopic images of Trypan blue stained Vero cells confirmed cytotoxicity response of STEC cells (Figure 2D). Furthermore, an increase in the concentration of STEC exposure increased dye uptake and cell rounding and cell damage.

**Table 1:** Bacterial cell populations in mTSB (USDA enrichment) after 15 h of growth.

Culture	Strain^a^	Serotype	PCR^b^	Cell density^c^
*Escherichia coli*	489	O157:H12	–	4.00 × 10^8^± 7.48 × 10^7^
	490	O157:H19	–	5.83 × 10^8^± 1.24 × 10^8^
	467	O5:NM	*stx1*	4.90 × 10^8^± 3.67 × 10^8^
	HSC7	O111:H8	*stx1*	5.00 × 10^8^± 5.35 × 10^7^
	SJ3	O26:H11	*stx2*	8.03 × 10^7^± 6.18 × 10^6^
	SEA13A72	O157:H7	*stx2*	2.77 × 10^8^± 1.11 × 10^8^
	488	O157:NM	*stx2*	4.07 × 10^9^± 1.81 × 10^9^
	HSC23	O91:H21	*stx2*	3.57 × 10^8^± 4.50 × 10^7^
	B1409-C1P	O157:H7	*stx2*	2.14 × 10^8^± 1.52 × 10^8^
	B1409-C1	O157:H7	*stx2*	1.37 × 10^8^± 5.14 × 10^7^
	505B	O157:H7	*stx1/2*	3.90 × 10^8^± 5.10 × 10^7^
	204P	O157:H7	*stx1/2*	4.57 × 10^8^± 1.15 × 10^8^
	EDL933	O157:H7	*stx1/2*	4.30 × 10^8^± 7.26 × 10^7^
	HSC27	O111:H8	*stx1/2*	6.63 × 10^8^± 1.08 × 10^8^
	HSC16	OR:H7	*stx1/2*	5.77 × 10^8^± 5.31 × 10^7^
	SJ12	O103:H11	*stx1/2*	6.37 × 10^7^± 9.74 × 10^6^
	SJ9	O45:H2	*stx1/2*	3.97 × 10^7^± 4.19 × 10^6^
	SJ18	O121:H19	*stx1/2*	4.46 × 10^8^± 2.86 × 10^8^
	SJ23	O145:NM	*stx1/2*	1.02 × 10^8^± 6.98 × 10^6^
*Salmonella enterica*	13ENT512	Heidelberg	NT	1.84 × 10^9^± 4.92 × 10^8^
	NA	Kentucky	NT	1.77 × 10^9^± 2.49 × 10^8^
	NA	Tennessee	NT	2.80 × 10^9^± 3.74 × 10^8^
*Listeria monocytogenes*	F4244	4b	NT	NG
*Listeria innocua*	F4248	NA	NT	NG
*Citrobacter freundii*	43864	NA	NT	2.37 × 10^1^± 6.34 × 10^2^
	8090	NA	NT	2.37 × 10^1^± 1.09 × 10^1^
*Serratia marcescens*	8100	NA	NT	3.20 × 10^3^± 2.16 × 10^4^
	43862	NA	NT	2.60 × 10^4^± 7.79 × 10^5^
*Hafnia alvei*	18066	NA	NT	2.37 × 10^4^± 5.25 × 10^5^


*^*a*^Strains were obtained from Auburn University, Food and Drug Administration, Agri-Food Canada, Center for Disease Control and Prevention and American Type Culture Collection, Dr. Pina Fratamico, and form our culture collection. ^*b*^*st*x*1* and *stx2* gene-based multiplex PCR was performed with initial denaturation at 95°C for 2 min, denaturation at 95°C for 1 min, annealing at 57°C for 1 min, and elongation at 72°C for 35 s. ^*c*^Isolates were plated on SMAC to enumerate bacterial cell density. NA, not available. NT, not tested. NG, no growth. –, negative for stx1 or stx2.*

Next, we determined an optimal time required to show a definite difference in cytotoxicity between *stx*^+^ (204P) and *stx^-^* (489) strains. At 2 h, there was no difference, while at 6 and 16 h post-infection (hpi), cytotoxicity values were found to be significantly (*P* < 0.05) different between *stx*^+^ and *stx^-^* strains (Figure 2E). Furthermore, the 3D Vero showed a higher response than the 2D Vero toward *stx*^+^ strain and consequent microscopic images confirmed this observation (Figure 2F).

### 3D Vero Cell Platform Is Specific for STEC Cells

We verified the sensitivity of the 3D Vero cell platform to 27 STEC and non-STEC strains at 2, 6, and 16 hpi. Data show that all STEC strains tested (including serovars O157, O26, O121, O103, O45, and O145) showed significantly higher cytotoxicity (*P* < 0.05) than the non-STEC strains at 6 and 16 h while no discernable cytotoxicity response at 2 h (Table 2). Since no significant difference was observed among negative controls at each time point on a 2D or 3D platform (*P* < 0.05), a cutoff value was determined by taking three standard deviations above the mean of all negative controls (Table 3), and the assay threshold is established to be 15% (Zhang et al., 1999; Moodie et al., 2010). Microscopic images of Vero cells captured after infection with *stx*^+^ STEC (204P) and non-STEC bacterial isolates (*Salmonella enterica* serovar Tennessee, *Salmonella* Heidelberg, *Salmonella* Kentucky, *Listeria innocua* F4248, *Listeria monocytogenes* F4244, *Citrobacter freundii*, *Serratia marcescens*, *Hafnia alvei*, and non-pathogenic *E. coli* isolates) were in agreement with the cytotoxicity data (Supplementary Figure S1).

**Table 2:** Cytotoxicity assay of STEC and non-STEC bacteria.

Culture	Isolate and serotype	Cytotoxicity (Abs_490/680_)^a^					
		
		2 h		6 h		16 h	
				
		2D (%)	3D (%)	2D (%)	3D (%)	2D (%)	3D (%)
	**48-well plate assay**						
	Low control	0.04 ± 0.01	0.05 ± 0.02	0.05 ± 0.02	0.05 ± 0.02	0.05 ± 0.01	0.05 ± 0.01
	(DMEM+LB)	(0%)	(0%)	(0%)	(0%)	(0%)	(0%)
	High control	0.74 ± 0.11	0.72 ± 0.19	0.69 ± 0.05	0.69 ± 0.21	0.71 ± 0.04	0.73 ± 0.08
	(2% Triton X-100)	(100%)	(100%)	(100%)	(100%)	(100%)	(100%)
*E. coli*	489 O157:H12	0.09 ± 0.01	0.06 ± 0.002	0.11 ± 0.02	0.09 ± 0.01	0.09 ± 0.02	0.07 ± 0.01
		(7%)	(0%)	(10%)	(7%)	(6%)	(3%)
	490 O157:H19	0.08 ± 0.01	0.07 ± 0.01	0.12 ± 0.05	0.13 ± 0.04	0.06 ± 0.004	0.11 ± 0.004
		(6%)	(2%)	(12%)	(13%)	(2%)	(9%)
	467 O5:NM	0.09 ± 0.01	0.07 ± 0.02	0.26 ± 0.01	0.44 ± 0.01	0.49 ± 0.03	0.56 ± 0.03
		(7%)	(2%)	(33%)	(61%)	(67%)	(75%)
	HSC7	0.07 ± 0.01	0.08 ± 0.02	0.24 ± 0.02	0.45 ± 0.08	0.22 ± 0.06	0.54 ± 0.06
	O111:H8	(4%)	(4%)	(30%)	(62%)	(26%)	(72%)
	SJ3 O26:H11	0.09 ± 0.004	0.07 ± 0.01	0.45 ± 0.01	0.54 ± 0.09	0.58 ± 0.01	0.68 ± 0.01
		(7%)	(2%)	(62%)	(76%)	(80%)	(92%)
	SEA13A72	0.09 ± 0.01	0.07 ± 0.03	0.32 ± 0.06	0.26 ± 0.01	0.29 ± 0.01	0.56 ± 0.01
	O157:H7	(7%)	(2%)	(43%)	(33%)	(37%)	(75%)
	488 O157:NM	0.15 ± 0.03	0.08 ± 0.02	0.38 ± 0.04	0.39 ± 0.09	0.36 ± 0.01	0.40 ± 0.01
		(15%)	(4%)	(51%)	(53%)	(47%)	(52%)
	HSC23	0.15 ± 0.03	0.08 ± 0.02	0.50 ± 0.10	0.56 ± 0.04	0.50 ± 0.02	0.60 ± 0.02
	O91:H21	(15%)	(4%)	(69%)	(79%)	(68%)	(81%)
	505B O157:H7	0.07 ± 0.05	0.10 ± 0.03	0.37 ± 0.02	0.54 ± 0.09	0.57 ± 0.01	0.63 ± 0.01
		(4%)	(7%)	(49%)	(77%)	(79%)	(85%)
	204P O157:H7	0.19 ± 0.03	0.17 ± 0.03	0.31 ± 0.03	0.43 ± 0.02	0.52 ± 0.01	0.57 ± 0.01
		(20%)	(17%)	(41%)	(60%)	(72%)	(76%)
	EDL933	0.06 ± 0.07	0.17 ± 0.03	0.26 ± 0.01	0.30 ± 0.01	0.46 ± 0.03	0.50 ± 0.03
	O157:H7	(3%)	(15%)	(32%)	(39%)	(62%)	(66%)
	HSC27	0.13 ± 0.07	0.15 ± 0.01	0.31 ± 0.03	0.42 ± 0.04	0.35 ± 0.01	0.62 ± 0.01
	O111:H8	(13%)	(4%)	(41%)	(57%)	(46%)	(84%)
	HSC16 OR:H7	0.08 ± 0.01	0.06 ± 0.001	0.50 ± 0.06	0.45 ± 0.07	0.45 ± 0.01	0.55 ± 0.01
		(5%)	(1%)	(70%)	(63%)	(60%)	(73%)
	SJ12	0.10 ± 0.004	0.08 ± 0.002	0.29 ± 0.02	0.43 ± 0.04	0.47 ± 0.02	0.67 ± 0.02
	O103:H11	(9%)	(3%)	(37%)	(59%)	(63%)	(91%)
	SJ9 O45:H2	0.09 ± 0.003	0.09 ± 0.003	0.30 ± 0.01	0.46 ± 0.07	0.48 ± 0.04	0.58 ± 0.04
		(7%)	(6%)	(38%)	(64%)	(66%)	(78%)
	SJ18	0.11 ± 0.01	0.07 ± 0.02	0.37 ± 0.02	0.45 ± 0.01	0.55 ± 0.02	0.64 ± 0.02
	O121:H19	(9%)	(2%)	(50%)	(62%)	(76%)	(86%)
	SJ23	0.04 ± 0.002	0.07 ± 0.16	0.23 ± 0.01	0.33 ± 0.04	0.36 ± 0.01	0.54 ± 0.01
	O145:NM	(0%)	(0%)	(28%)	(44%)	(47%)	(72%)
*Salmonella*	Heidelberg	0.05 ± 0.01	0.06 ± 0.02	0.05 ± 0.01	0.05 ± 0.002	0.07 ± 0.001	0.07 ± 0.001
	13ENT512	(2%)	(1%)	(1%)	(1%)	(2%)	(2%)
	Kentucky	0.05 ± 0.002	0.06 ± 0.002	0.05 ± 0.01	0.07 ± 0.01	0.10 ± 0.004	0.07 ± 0.004
		(1%)	(1%)	(1%)	(3%)	(7%)	(3%)
	Tennessee	0.05 ± 0.002	0.05 ± 0.01	0.06 ± 0.001	0.06 ± 0.01	0.09 ± 0.001	0.05 ± 0.001
		(1%)	(0%)	(2%)	(2%)	(6%)	(0%)
*Listeria*	F4244	0.06 ± 0.01	0.05 ± 0.02	0.04 ± 0.01	0.05 ± 0.001	0.13 ± 0.002	0.09 ± 0.002
*monocytogenes*		(3%)	(0%)	(0%)	(1%)	(12%)	(5%)
*Listeria*	F4248	0.04 ± 0.001	0.06 ± 0.003	0.10 ± 0.03	0.05 ± 0.01	0.13 ± 0.002	0.08 ± 0.002
*innocua*		(0%)	(0%)	(8%)	(0%)	(11%)	(5%)
*Citrobacter*	43864	0.07 ± 0.02	0.05 ± 0.002	0.05 ± 0.002	0.06 ± 0.03	0.08 ± 0.002	0.09 ± 0.03
*freundii*		(3%)	(0%)	(0%)	(2%)	(5%)	(6%)
	8090	0.08 ± 0.01	0.06 ± 0.01	0.05 ± 0.003	0.06 ± 0.01	0.07 ± 0.003	0.10 ± 0.01
		(5%)	(1%)	(0%)	(2%)	(3%)	(7%)
*Serratia*	8100	0.06 ± 0.001	0.09 ± 0.03	0.11 ± 0.08	0.07 ± 0.03	0.09 ± 0.08	0.06 ± 0.03
*marcescens*		(3%)	(6%)	(10%)	(4%)	(7%)	(1%)
	43862	0.07 ± 0.01	0.07 ± 0.03	0.13 ± 0.10	0.07 ± 0.03	0.08 ± 0.10	0.08 ± 0.03
		(3%)	(3%)	(13%)	(3%)	(4%)	(4%)
*Hafnia alvei*	18066	0.06 ± 0.001	0.06 ± 0.003	0.05 ± 0.004	0.05 ± 0.01	0.09 ± 0.004	0.09 ± 0.01
		(2%)	(1%)	(0%)	(1%)	(7%)	(6%)


*^*a*^Percent cytotoxicity was calculated using the formula: [(A_*Exp*_ - A_*LB control*_)/(A_*Triton X*_ - A_*LB control*_)] × 100 based on the amount of LDH released from negative control, positive control, and experimental samples; percent cytotoxicity is an average of three experiments displayed as means (±SD).*

**Table 3:** Stx yield from *stx*^+^ 204P 5 h after induction with UV, mitomycin C, ciprofloxacin, or polymyxin B.

Treatment	Vol. (ml)	Un concentrated (total protein μg/ml)^e^	Max. Stx yield		10 × concentrated (total protein μg/ml)^e^	Max. Stx yield	
					
			Stx1	Stx2		Stx1	Stx2
Control	50	723 ± 99	ND	ND	996 ± 317	ND	ND
	200	3814 ± 82	ND	ND	3984 ± 317	ND	2.75 ± 0.01
UV^a^	50	1010 ± 119	ND	215 ± 7.5	1533 ± 42	316 ± 475	1687 ± 0.42
Mitomycin C^b^	50	1766 ± 87	72.7 ± 32	81.3 ± 0.12	1915 ± 10	906 ± 305	3298 ± 0.30
Ciprofloxacin^c^	50	1826 ± 35	852 ± 7.5	10.66 ± 0.01	2738 ± 612	1260 ± 12	314 ± 0.04
Polymyxin B^d^	200	3314 ± 314	ND	ND	3540 ± 0.108	ND	417 ± 0.06


*^*a*^UV lamp was placed 12 inches away from samples and treated at 115 V/68 Amps for 1 m before further incubation at 37°C for 5 h. ^*b*^Mitomycin C (2 μg/ml) added to overnight cultures diluted 1:50 after 3 h incubation at 37°C before further incubation at 37°C for 5 h. ^*c*^Ciprofloxacin (100 ng/ml) added to overnight cultures diluted 1:50 after 3 h incubation at 37°C before further incubation at 37°C for 5 h. ^*d*^Polymyxin B (1 mg/ml) added to pellet from overnight culture and incubated at 37°C for 30 min. ^e^Total protein quantified using BSA curve, *y* = 0.001069*x* + 0.05491.*

### Antibiotic Induction of STEC Increases the Sensitivity of 3D Vero Cell Platform

**FIGURE 3:**
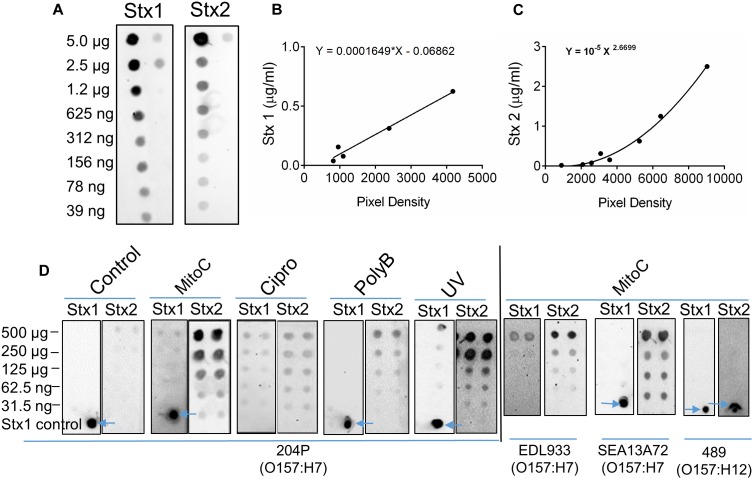
Antibiotic induction of STEC increases toxin production. **(A)** Twofold serial dilutions of commercial Stx1 and Stx2 obtained from Toxin Technologies (Sarasota, FL, United States) were used to establish toxin standard curves **(B,C)**, respectively. **(D)** Dot blot analysis of crude Stx1 and Stx2 protein concentrated from *stx*^+^ strains (204P, EDL933, SEA13A72), and *stx*^-^ (489) 5 h after Stx induction using antibiotics or UV light. Commercial Stx1 was used as a control in some blots (see arrows).

**FIGURE 4:**
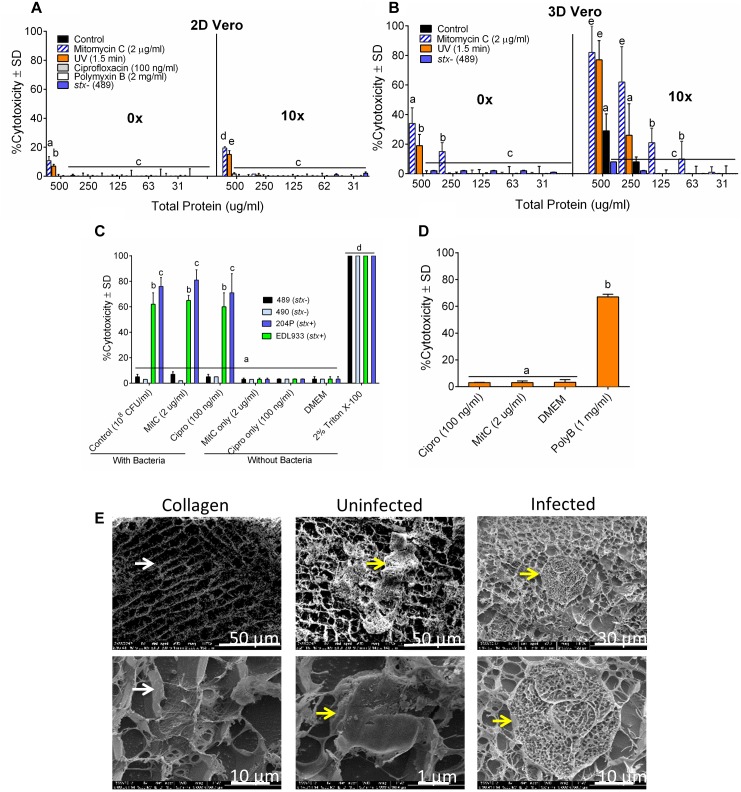
The sensitivity of 3D Vero cell platform. Cytotoxicity response of toxin preparations (0 or 10× concentrated) from *E. coli* strain 204P (*stx*^+^) after antibiotic or UV induction on **(A)** 2D Vero and **(B)** 3D Vero after 16 h exposure. **(C)** Vero cells exposed to bacteria pre-induced with antibiotics: STEC and non-pathogenic *E. coli* and **(D)** antibiotics alone: ciprofloxacin (Cipro), mitomycin C (MitC), and polymyxin B (Poly B) for 3 h and 16 h, respectively, at 37°C in 5% CO_2_. **(E)** Cryo-SEM images of the collagen embedded Vero cells exposed to crude Stx preparation from mitomycin C induced *E. coli* strain 204P (O157:H7) incubated for 8 h.

Our ultimate goal is to use an Stx-induction agent, such as antibiotics, together with the traditional bacterial enrichment broth before testing of food samples on a 3D cell-based assay platform without an intermediate sample-processing step. Antibiotics-induced Stx production has been reported before (Yee et al., 1993; Shimizu et al., 2003). Therefore, we wanted to examine the effect of several antibiotics, such as mitomycin C (2 μg/ml), ciprofloxacin (100 ng/ml), polymyxin B (2 mg/ml) and ultraviolet light (UV, exposure time 1.5 min) on Stx production and corresponding cytotoxicity on 2D and 3D Vero cell platforms. Data show that mitomycin C and UV induced the highest amount of Stx production than the ciprofloxacin or polymyxin B (Table 3). Toxin production was quantified in a dot blot immunoassay using anti-Stx1 and anti-Stx2 antibodies, and commercial Stx1 and Stx2 toxins as standards (Figure 3). Mitomycin C and UV induced 316 ± 475–906 ± 305 μg/ml Stx1 and 1687 ± 0.42–3298 ± 0.30 μg/ml Stx2, respectively. Ciprofloxacin induced 1,260 ± 12 μg/ml Stx1 and 314 ± 0.04 μg/ml Stx2 while polymyxin B induced undetectable levels of Stx1 and 417 ± 0.06 μg/ml Stx2. The crude toxin preparations (10× concentrated or unconcentrated) were then tested against 2D and 3D Vero cell platforms, and both showed positive effects. Notably, the effect was significantly higher on 3D than the 2D Vero cells (Figure 4A,B).

A 3-h pre-induction of *stx*^+^ strains (EDL933 and 204P) with mitomycin C (2 μg/ml) yielded 65 ± 4% and 81 ± 8% cytotoxicity, respectively. A similar trend was observed for ciprofloxacin, achieving 60 ± 11% and 71 ± 15% cytotoxicity, respectively, for these two strains. For uninduced bacteria, exposure to the same isolates had cytotoxicity values of 62 ± 9% and 76 ± 7%, respectively (Figure 4C). As expected, *stx^-^* strains (489 and 490) showed negligible cytotoxicity (3–7 ± 2%) with or without antibiotics. We also tested the cytotoxic effects of all antibiotics, if any, without the bacteria and only polymyxin B caused cytotoxicity on Vero cells (Figure 4D). Cryo-scanning electron microscopy (SEM) images also showed cell damage induced by Stx preparation from 204P strain after mitomycin C induction (Figure 4E). Stx-induced cell damage is mostly characterized by porous honeycomb-like cell architecture. Collectively, these data indicate that mitomycin C, ciprofloxacin and the UV treatment induced Stx production, and the cell-free toxin preparation showed a significantly higher cytotoxic effect than the toxin preparation from the uninduced cells. However, differences in cytotoxicity were not significant for STEC cells with or without induction when tested against the 3D Vero cells; therefore, antibiotics were not used with our food sample testing experiment described below.

### STEC Detection From Inoculated Beef Samples Using 3D Vero Cell

**FIGURE 5:**
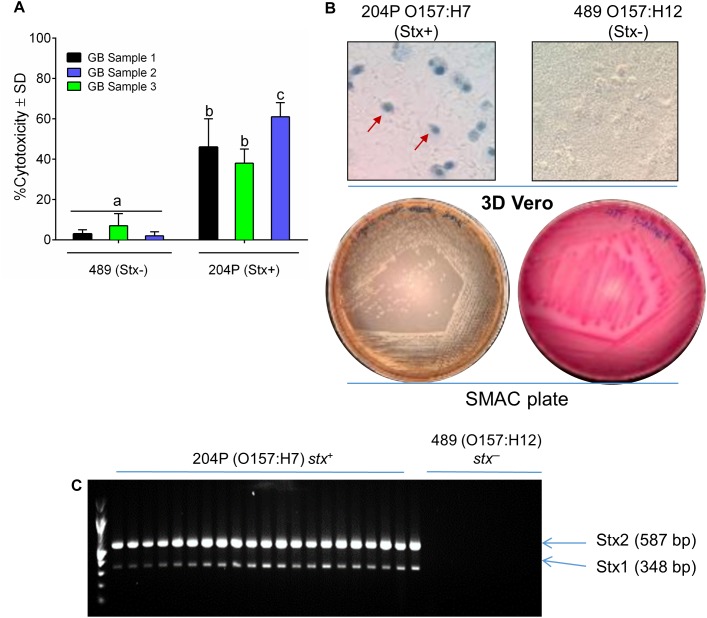
Detection of STEC from inoculated ground beef samples using 3D Vero. **(A)** Percent cytotoxicity induced by STEC from inoculated ground beef samples (*n* = 3 × 9 = 27) on 3D Vero. **(B)** Trypan blue staining of treated Vero cells shows cell rounding and membrane damage after exposure to ground beef spiked with strain 204P (*stx*^+^) but not with 489 (*stx*^-^). Plating on SMAC plate **(B)** and PCR **(C)** confirmed the presence of STEC cells and *stx1* (348 bp) and *stx2* genes (587 bp), respectively, from inoculated ground beef samples.

*Escherichia coli* 204P (*stx*^+^) and 489 (*stx^-^*) – inoculated raw ground beef samples (3 samples × 9 technical replicates = 27), procured from local grocery stores were enriched in mTSB at 42°C for 15 h (FDA, 2001). An aliquot of the enriched food samples was centrifuged and resuspended in LB and then applied to 3D Vero cell platform and incubated for 6 h. Note, mTSB alone exhibited cytotoxic response against Vero, and thus this medium was replaced with LB before Vero assay (Supplementary Figure S1). A 15-h enrichment time was previously determined to yield a cell concentration of at least 10^7^ CFU/ml (Figure 2 and Table 1) required for positive cytotoxicity on the 3D Vero platform. The 3D Vero cell assay positively detected STEC form all artificially contaminated ground beef samples tested with cytotoxicity values varied from 36 to 61% (Figure 5A). Strain 489 (*stx^-^*) served as a negative control for the assay and yielded 3–7% cytotoxicity, which is significantly lower than the *stx*^+^ samples (*P* < 0.05). Trypan blue staining of Vero cells confirmed dye uptake, cell rounding and characteristic cytotoxic effect, which was in good agreement with LDH-based cytotoxicity assay (Figure 5B). The presence of STEC in the enrichment broth (mTSB) was re-confirmed after subculturing on SMAC plates and multiplex PCR analysis of colonies for the presence of *stx1* and *stx2* genes (Figure 5C). These data clearly indicate that the 3D Vero cell platform is suitable for the detection of STEC cells from raw ground beef samples.

## Discussion

This study demonstrates the application of the 3D Vero cell platform for rapid and sensitive detection and screening of Shiga-toxin producing *E. coli* from non-STEC bacteria in food samples. We employed the 3D Vero cell platform, which was able to detect both STEC cells and cell-free toxins in 6 h. This 3D Vero cell platform showed a cytotoxic response only to STEC cells or Stx preparations but no response to non-STEC cells. In this study, the Vero cell culturing in 3D platform, and toxin induction approach helped improve the assay sensitivity thereby reducing the detection time to about 6 h, which is a significant improvement over previous reports (Konowalchuk et al., 1977; Roberts et al., 2001; Quiñones et al., 2009). We found that the Vero cells in the 3D platform are more sensitive to STEC than the 2D setup. This may be primarily attributed to the 3D scaffold mimicking tissues in the body by maintaining cell polarity thus providing greater access of Vero cell surface receptors to pathogens and toxins (Ravi et al., 2015; Barrila et al., 2018). Furthermore, the collagen embedding provides stable support for Vero cells during testing of food samples without any possibility of loss of cells, which can happen when the cells are grown in 2D monolayers. Additionally, collagen’s strong gelling properties can act as a protective barrier to enhance the viability of cells and eliminate the risk of tampering during field-deployment (Banerjee et al., 2008; Parenteau-Bareil et al., 2010). Previously, a similar 3D cell culture system using Ped-2E9 hybridoma B cells significantly improved toxin detection from *Listeria monocytogenes*, and *Bacillus cereus* (Banerjee et al., 2008; Banerjee and Bhunia, 2010). Overall, a 6 h 3D Vero cell-based assay is more competitive than the previously modified 2D Vero cell-based assay (12–16 h) (Roberts et al., 2001), and compatible with immunoassays (Tokarskyy and Marshall, 2008; Il-Hoon et al., 2015; Wang et al., 2016; Armstrong et al., 2018), PCR (Cui et al., 2003; Amani et al., 2015; Parsons et al., 2016), and light scattering sensor (Tang et al., 2014), thus provides an attractive alternative approach for the screening of food samples for the presence of STEC.

Antibiotics or phage induction methods are typically used to induce or release Stx from STEC bacteria either to improve Stx yield for pathogenesis studies or to improve sensitivity for detection assays (Griffin and Gemski, 1983; Yee et al., 1993; Zhang et al., 2000; Laing et al., 2012; Armstrong et al., 2018). As Stx prophages tightly regulate Stx production and release, activation of the SOS response gene, *recA*, must occur to express downstream *stx1* and *stx2* genes (Tyler et al., 2013). DNA damaging agents such as mitomycin C and ciprofloxacin that inhibit DNA synthesis or topoisomerase I, respectively, can induce bacterial SOS response and induce Stx production. Therefore, mitomycin C, ciprofloxacin, and polymyxin B were used at recommended concentrations (Roberts et al., 2001; Shimizu et al., 2003; Aertsen et al., 2005; Maldonado et al., 2005; Wang et al., 2016). Interestingly, Vero cells showed a strong cytotoxic response to polymyxin B (1 mg/ml) in absence of bacterial toxins. Polymyxin B mediated cytotoxic event is attributed to the interaction of polymyxin B with the cytoplasmic membrane of mammalian cells while disrupting osmotic homeostasis and promotion of oxidative stress (Neiva et al., 2013; Vattimo et al., 2016) thus, polymyxin B was not used in subsequent experiments. Furthermore, STEC pre-induced with mitomycin C and ciprofloxacin for 3 h did not improve the sensitivity of Vero cells to STEC suggesting that antibiotics may not be necessary for the development of 3D Vero assay for detection of STEC cells from food samples. STEC pathogenesis is multifactorial involving bacterial intimate attachment and production of attachment effacement lesion, which is substantiated by upregulation of genes in LEE, activation of T3SS, and Stx production for a cytotoxic response (Schüller, 2011; Tran et al., 2014; Licznerska et al., 2016; Bhunia, 2018). This may explain why Stx-mediated additive cytotoxic response after antibiotic induction was not seen when used with the STEC cells. However, mitomycin C and ciprofloxacin induction may yield a higher Stx amount during toxin preparation and for enhanced cytotoxicity response (Yee et al., 1993; Schüller, 2011).

An enrichment step is essential for food testing as it ensures full recovery and increased bacterial numbers without running the risk of generating false negatives (Sharpe, 2001; Bhunia, 2014; Vetter, 2016). Although the detection limit of 3D Vero cell is high, requiring at least 10^7^ CFU/ml, a 15-h enrichment in mTSB should be able to achieve 10^7-8^ CFU/ml in the test sample with a very low (<100 CFU/25 g) initial inoculum for reliable detection of STEC from food samples. This enrichment step for sample preparation is required for most, if not all detection assays including immunoassay and PCR (Gould et al., 2009). It is well documented that PCR and immunoassays have superior sensitivity in detecting STEC (10 CFU/ml) and Stx (0.5 ng/ml) (Il-Hoon et al., 2015; Gehring et al., 2017); however, there are also drawbacks in detection time, sample and reagent preparation, and information provided about functional activity of Stx and viability of STEC (Pimbley and Patel, 1998; Cui et al., 2003; Banerjee et al., 2013; Amani et al., 2015; Zeng et al., 2016). Vero cell assay can also detect picogram quantities of Stx, however, detection time must be extended to 24–72 h to acquire confirmatory results (Hughes et al., 1998; Rasooly and Do, 2010; Lentz et al., 2011). We verified that the detection time plays a role in the sensitivity of the assay using commercial Stx1 and Stx2 toxins preparations. Additionally, crude Stx sample preparation with the lengthy isolation, enrichment, and concentration methods makes it cumbersome to be used as an analyte for routine rapid detection.

To overcome the burden of sample and assay preparation with a faster sampling and detection time, 3D Vero cell system has been validated with inoculated ground beef samples for successful detection of viable STEC cells in 6 h after an enrichment step. In the 3D Vero cell assay, LDH release from toxin-induced cell death was measured as an indicator for cytotoxicity. However, this enzyme is found in animal tissues (meat), so interference from ground beef samples before inoculation with STEC or non-STEC cells must be calculated to correct for final values (Collins et al., 1991; Šalplachta and Nečas, 2000). Therefore, appropriate controls must be included every sampling time while using the 3D Vero platform. Furthermore, *Salmonella, Listeria*, *Citrobacter*, *Serratia*, *Hafnia*, and non-pathogenic *Escherichia coli* following a 15-h enrichment in mTSB did not yield positive cytotoxicity results, confirming the specificity of 3D Vero to detect STEC. Additionally, the purpose of using non-pathogenic *E. coli*, untreated mammalian cells, enrichment media, and antibiotics as negative controls throughout the study is to evaluate the level of interference that could affect the performance of the assay. With a low interference level, which allowed us to establish a 15% cutoff value, reduce the risk of achieving false positive results.

In summary, we demonstrated that the 3D Vero cell-based assay could be used for rapid detection of STEC by measuring LDH release. During method development, sample preparation time was shortened by using a bacterial analyte rather than a toxin analyte where the use of antibiotics can be eliminated in the enrichment step. Antibiotic-uninduced STEC resuspended in LB exhibited desirable cytotoxicity levels (26–81%) that is above the 15% threshold after 6 h post-infection of the 3D Vero cell. Concentrated culture filtrates after mitomycin C induction also induced comparable levels of cytotoxicity, which was validated with dot immunoblotting showing high Stx2 yield (3.2-fold). Despite the high Stx1 production from ciprofloxacin induction (1.4-fold), low Stx2 yield was achieved. Mitomycin C was chosen for further studies due to the level of importance of Stx2 in STEC infection and its association with HUS. When compared to control cells, Vero cell morphology was profoundly altered in response to viable STEC cells and active Stx observed after Trypan blue staining and Cryo-SEM. Vero cells in either 2D or 3D configuration can detect up to 10^7-8^ CFU/ml or 1,000 ng/ml (2D) and 31.25 ng/ml (3D) of Stx in 6 h, respectively. THP-1 cells did not exhibit a strong positive signal against STEC cells as compared to the Vero cells but can detect up to 125 ng/ml of Stx and 31.25 ng/ml of Stx in a 2D and 3D configurations, respectively. Since 3D Vero cells performed the best in response to both STEC cells and crude Stx, the 3D Vero platform was successfully used for the screening and detection of STEC cells from artificially contaminated ground beef samples in 6 h, which is faster than the traditional gold standard Vero cell assay which takes about 72-h. This method provides an opportunity to screen for emerging STEC serotypes with similar cytotoxicity potential. Therefore, this method for screening viable STEC has the potential to be adopted by the public health and/or food industry sectors for the screening of STEC from various samples to prevent future occurrence of STEC related foodborne outbreaks.

## Materials and Methods

### Bacterial Cultures and Growth Media

Frozen stock cultures (Table 1) were grown in BHI (Brain Heart Infusion) broth and then maintained on BHI agar (BHI, Becton Dickinson) plate for 1 month at 4°C. For fresh cultures, isolated colonies were inoculated into modified tryptone soy broth (mTSB) containing 0.15% bile salt, 0.4% dipotassium hydrogen phosphate, and 0.25% glucose (Becton Dickinson, Franklin Lakes, NJ, United States) at 42°C for 15 h with shaking at 120 rpm. Bacteria were also grown in Luria Bertani (LB) broth (10 g/L tryptone, 5 g/L yeast extract, 10 g/L NaCl), and EC broth (Becton Dickinson). To confirm and verify cultures, isolates were streaked on Modified Oxford Agar (MOX, Neogen, Lansing, MI, United States), Xylose Lysine Tergitol 4 (XLT4, Becton Dickinson), Sorbitol McConkey Agar (SMAC, Becton Dickinson) and RAPID’Enterobacteriaceae Medium (Bio-Rad, Hercules, CA, United States).

### Mammalian 2D and 3D Cell Culture

African green monkey kidney (Vero) cell line (ATCC CCL-81) were purchased from the American Type Culture Collections (ATCC) and maintained in Dulbecco’s modified Eagles medium (DMEM) (Sigma, St. Louis, MO, United States) with 10% fetal bovine serum. For all cytotoxicity assays, 3.2 × 10^4^ Vero or undifferentiated THP-1 (human monocyte cell line, ATCC) cells were grown as monolayers (2D) or suspensions (2D), respectively, in 48 well plates at 37°C with 5% CO_2_ under humidity for 24 h. Vero cell monolayers were trypsinized with 0.25% of trypsin (Sigma) as described by the vendor and cell counts were determined by Trypan blue (0.4%) staining (Sigma). For 3D cell culture, cell suspensions were centrifuged and about 3.2 × 10^4^ Vero or undifferentiated THP-1 cells were embedded with collagen (0.7 mg/ml) containing 50 μl PBS, 113 μl collagen I, 2.5 μl NaOH, and 335 μl DMEM (Sigma) as described (Banerjee et al., 2008) in 48 well plates (Corning). DMEM or RPMI supplemented with 10% fetal bovine serum (FBS) was added after a 30-min incubation at 37°C in 5% CO_2_ to allow for complete gelation.

### Cytotoxicity Assay

Lactate dehydrogenase release was measured as described before (Roberts et al., 2001; Maldonado et al., 2005). THP-1 suspension cells were centrifuged (1,800 × *g* for 3 min), washed with serum-free RPMI and seeded at 3.2 × 10^4^ cells in 48 well plates with an initial volume adjusted to 200 μl/well. Vero cell monolayers and Vero embedded cells were washed with serum-free DMEM before the addition of DMEM (200 μl/well). Cells were exposed to 300 μl of STEC cells (∼10^8^ CFU/ml) for 2 to 16 h or serial dilutions of crude or commercial Stx1a, Stx2a, and Stx2c toxins (Toxin Technologies, Sarasota, FL, United States or BEI Resources, Manassas, VA, United States) for 16 h. Vero or THP-1 cell supernatants were collected after centrifugation (1,800 × *g* for 3 min) and loaded onto a sterile 96 well plate. Samples (50 μl) were mixed with LDH reaction reagent (50 μl) containing diaphorase, NAD^+^, sodium lactate, and iodophenyl-nitrophenyl-phenyltetrazolium chloride (Pierce Biotechnology, Rockford, IL, United States). Plates were incubated for 15–20 min in the dark at room temperature before taking measurements at absorbance 490/680 nm for LDH release using a microplate reader (Epoch, BioTek, Winooski, VT, United States). High controls treated with Triton X-100 (2%) for 45 min and low controls treated with serum-free DMEM+LB were used to calculate the percent cytotoxicity. Crude toxin preparations, STEC cells (204P) or commercial Stx toxins were used as positive controls for all LDH assays while cell-free supernatant or cells from *stx*^-^ strain (O157:H12 strain 489) were used as negative control. To visualize cytopathic effect from treatments, cells were fixed with formaldehyde (4% in PBS, Sigma) and stained with Trypan blue (4%, 1:2 dilution, Corning, Waltham, MA, United States) solution for 3 min. Images were captured at 400× magnification using an Olympus Inverted Microscope.

### Effect of Growth Media on Cytotoxicity

Four different growth media were evaluated to test for potential interference with the assay. Three hundred microliters of each LB broth, EC broth, and DMEM were added to 200 μl of DMEM before exposure to Vero cells and incubated for 16 h at 37°C in 5% CO_2_. LDH assay of cell supernatants was performed as above.

### Stx Induction by Antibiotics and UV

For antibiotic induction, Vero cells were exposed to 500 μl of DMEM containing polymyxin B (50 μg/ml, Sigma), mitomycin C (24 ng/ml, Sigma), or ciprofloxacin (1.2 ng/ml, Sigma) for 16 h at 37°C in 5% CO_2_. To verify the activity of antibiotics, Vero cell supernatants were tested against EDL933 (10^8^ CFU/ml) on BHI. Since polymyxin B can potentially interfere with the assay and can give a high background signal, mitomycin C and ciprofloxacin were used for further experiments. Since LB and DMEM had the least cytotoxicity response, these two media were then used as resuspension media to evaluate cytotoxicity response of *stx*+ positive and *stx*^-^ samples after a 3 h pre-induction with mitomycin C (2 μg/ml) and ciprofloxacin (100 ng/ml) at 37°C with shaking at 120 rpm. All samples were analyzed for LDH release after 16 h as described above. Vero cells were imaged under light microscopy (Olympus) at 400× magnification.

### Cell-Free Toxin Preparations

Crude toxins were prepared from *stx*+ O157:H7 strains (204P, EDL933, SEA13A72), O5:NM (467), and *stx*^-^ O157:H12 strain 489. Bacteria were grown in mTSB at 37°C for 15 h with shaking at 120 rpm. An aliquot of each culture was centrifuged and re-suspended to a final volume of 300 μl in LB to reach a concentration of 10^8^ CFU/ml and used immediately for LDH assay as above.

Overnight cultures were diluted to 1:50 in 50 ml LB and incubated for 3 h at 37°C with shaking at 120 rpm before the addition of mitomycin C (2 μg/ml) or ciprofloxacin (100 μg/ml, Sigma). For UV light treatment, overnight cultures were pelleted at 10,000 × *g* for 3 min, resuspended in LB, and dispensed into a sterile petri dish which was placed under a UV lamp (115 V, 68 Amps, UVP, Upland, CA, United States) for 1 min maintaining a distance of 12 inches between the lamp and sample. All cultures were further incubated for 5 h at 37°C with shaking at 120 rpm before collecting supernatant after centrifugation at 10,000 × *g* for 3 min. For polymyxin B treatment, 50 ml overnight culture was pelleted, re-suspended in 500 μl of PBS, supplemented with polymyxin (1 mg/ml), and further incubated for 30 min before collecting supernatant after centrifugation at 10,000 × *g* for 3 min. Pellets from all treatments were incubated with 100 μl of chloroform at room temperature for 30 min to improve Stx release and toxin yield. Cell-free supernatants of all treatments were concentrated to about 3–5 ml (about 10-fold concentrated) using Amicon Ultra-15 Centrifugal Filter Units using a 50 kDa cut-off cellulose membrane at 5,000 × *g* for 15 min (Millipore Sigma, Billerica, MA, United States).

### Dot Blot Analysis

Nitrocellulose membranes (0.2 μm, Biotrace NT, Thermo Scientific, Rochester, NY, United States) were pre-wetted in Tris-buffered saline with 0.1% tween 20 (TBST) for 1 min and placed in Bio-Dot apparatus (Bio-Rad, Hercules, CA, United States) under vacuum. Twofold diluted crude toxin samples and commercial Stx standards (Toxin Technologies, Sarasota, FL, United States, or BEI Resources, Manassas, VA, United States) were loaded into each well and application of gentle vacuum allowed fluid passage through the membrane. Membranes were washed with 100 μl of TBST/well, blocked with TBST containing 5% non-fat dry milk (NFDM), and incubated at room temperature for 45 min. Membranes were then washed with TBST three times at room temperature for 3 min with gentle agitation. Primary antibody solution containing antibody to Stx1-1 or Stx2-5 (Skinner et al., 2014, 2015; Wang et al., 2016) at dilutions 1:1000 in TBST with 5% NFDM was added to blots and allowed to incubate overnight at 4°C.

### Evaluation of Cytotoxicity Response From 2D and 3D Vero Cell

Twenty-seven bacterial isolates were screened for cytotoxicity potential after a 15 h enrichment in mTSB at 42°C with shaking. Vero cells (2D and 3D) were washed with serum-free DMEM (SF-DMEM) before exposure to bacteria (300 μl ≈ 10^8^ CFU/ml). The total volume of wells was adjusted to 500 μl with SF-DMEM before incubation at 37°C in 5% CO_2_. To determine the limit of detection of the assay, overnight cultures of *stx*^+^ (204P) and *stx*^-^ (489) were resuspended in LB and serial dilutions of bacterial preparations were added to Vero cells and incubated for 16 h at 37°C in 5% CO_2_. To determine optimal detection time, Vero cell cytotoxicity was assayed after exposure to *stx*^+^ (204P) and *stx*^-^ (489) at 10^8^ CFU/ml) for 2 h, 6 h, and 16 h. *Salmonella*, *Listeria* spp., *Citrobacter*, *Hafnia*, and *Serratia* spp. were also tested to investigate assay specificity. All samples were centrifuged at 1,800 × *g* for 3 min and assayed for LDH. LDH values were acquired from three independent sets of experiments and analyzed in duplicates. After LDH analysis, cells were fixed with formaldehyde (4%) before Trypan blue (4%, 1:2 dilution) staining for 3 min to determine Vero cell morphology after treatments at 400× magnification.

### Cryo-SEM of Vero Cells in 3D Matrix

Minimally dehydrated collagen gel embedded Vero cells were mounted on slotted Gatan holders with 1 mm of the sample above the holder. Samples were cryo-transferred after plunge freezing with nitrogen slush into the Gatan preparation chamber, which is held under high vacuum. Frozen samples were fractured with a cold scalpel in the Gatan Alto 2500 preparation chamber at -185°C (Pleasanton, CA, United States) before being transferred into the SEM chamber. Samples were mounted on the cryo-stage set for -90°C sublimation and imaged until a structure was observed before sputter coating for 120 s using a platinum target at -185°C. SEM Cryo-stage was lowered to -140°C during sputter coating and was reinserted onto the NovaNano SEM cryo-stage before capturing final images of fractured surfaces. An accelerating voltage of 5 kV and spot size 3 with a working distance of approximately 5 mm at magnifications ranging ×1,000 and ×10,000 were used in the NovaNano SEM.

### STEC Detection From Artificially Contaminated Ground Beef Using 3D Vero

3D Vero cell platform was used to screen and detect STEC from artificially contaminated ground beef samples. Three brands of ground beef (5 lb each), purchased from local grocery stores, were artificially inoculated with *stx*^+^ 204P (O157:H7) and *stx*^-^ 489 (O157:H12) strain under aseptic conditions. Samples were processed following the USDA guidelines for screening STEC in ground beef samples (FSIS, 2016). In brief, ground beef samples (32 ± 3.2 g each, 3 samples × 9 technical replicates = 27) were placed in sterile strainer bags (Seward, Islandia, NY, United States) inoculated with 100 μl of 204P (6.7 × 10^2^ ± 2.1 × 10^2^ CFU/ml) and 489 (7.4 × 10^2^ ± 5.9 × 10^2^ CFU/ml). Samples were then diluted with 97 ± 1.9 ml of mTSB and hand massaged for 1 min to homogenize before incubating at 42°C for 15 h with shaking at 120 rpm. To determine a final cell density after enrichment, samples were serially diluted (1:10 in PBS) and plated on SMAC plates and incubated at 37°C for 24 h. For 3D Vero cell assay, 300 μl samples were centrifuged at 10,000 × *g* for 3 min and resuspended in the same volume of LB before exposure to 2D or 3D Vero cells (3.2 × 10^4^ cells) in a 48-well plate format. After incubation at 37°C for 6 h in 5% CO_2_, samples were centrifuged at 1,800 × *g* for 3 min and analyzed for LDH release. Uninoculated meat samples were also tested to check for background response and interference. Suspected colonies identified on the SMAC were confirmed by PCR, targeting *stx1* and *stx2* genes.

### DNA Extraction and Multiplex PCR

DNA templates were extracted following a boiling and colony PCR method (Feng et al., 2002; Hoang Minh et al., 2015). Stx-specific primers targeting *stx1* (5′-ATCCTATTCCCGGGAGTTTACG-3′ and 5′-GCGT CATCGTATACACAGGAGC-3′) and *stx2* (5′-CACCAGACAATGTAACCGCTG-3′ and 5′-TIACCATTTCAGTACCTTCTGGTAA-3′) genes were used to confirm the Stx presence in the 3D Vero-identified STEC positive samples. DNA templates from non-STEC isolates, uninoculated meat samples, and water were used as negative controls. The reaction mixture (25 μl) contained 200 ng of DNA template, 1× GoTaq Flexi buffer, 1 U of GoTaq, 2.5 mM MgCl_2_, 200 μM deoxynucleoside triphosphates (dNTP), and 4 μM of *stx1* and *stx2* forward and reverse primers. The PCR was carried out with an initial denaturation step at 94°C for 120 s, followed by 35 cycles of denaturation at 95°C for 60 s, annealing at 57°C for 60 s, 72°C for 60 s and a final extension at 72°C for 35 s.

### Statistical Analysis

Values are presented as mean ± SD. GraphPad Prism (version 6) software was used to perform ordinary one-way or two-way ANOVA followed by Tukey’s multiple comparisons analysis to evaluate whether there are a significant difference among media (LB, DMEM, EC, mTSB) and antibiotics (mitomycin C and ciprofloxacin) induced cytotoxicity response. ImageJ (NIH) was used to assess Stx yield after induction. SAS software was used to perform 2 × 2 × 3 ANOVA followed by Tukey’s *post hoc* analysis to assess the interaction between *stx*^+^ and *stx*^-^ strains, dimension (2D vs. 3D), and time of incubation (2 h, 6 h, and 16 h).

## Author Contributions

AB conceived the idea. AB and CT designed the experiments, analyzed the data, and wrote the manuscript. CT performed the experiments.

## Conflict of Interest Statement

The authors declare that the research was conducted in the absence of any commercial or financial relationships that could be construed as a potential conflict of interest.

## Supplementary Material

Figure S1 Specificity assay. **(A)** Effect of growth media (DMEM, LB, EC, mTSB, *stx*^-^) on Vero cytotoxicity. **(B,C)** Cytotoxicity of non-STEC bacteria on 3D Vero cells after 6 h exposure. **(C)** STEC strain EDL933 caused severe cell damage, cell rounding, and cell death visualized after Trypan blue staining while other pathogens (*Salmonella* Tennessee, *Listeria monocytogenes*, *Serratia marcescens*, *Citrobacter freundii*, *Hafnia alvei*, and *Escherichia coli*) did not cause any damage during this period (magnification, 400×).
