# Supplementary figures and images for: Three Dimensional Vero Cell-Platform for Rapid and Sensitive Screening of Shiga-Toxin Producing Escherichia coli
Source: Front Microbiol. 2019 May 7;10:949. doi: 10.3389/fmicb.2019.00949 (PMC6514307; doi:10.3389/fmicb.2019.00949)

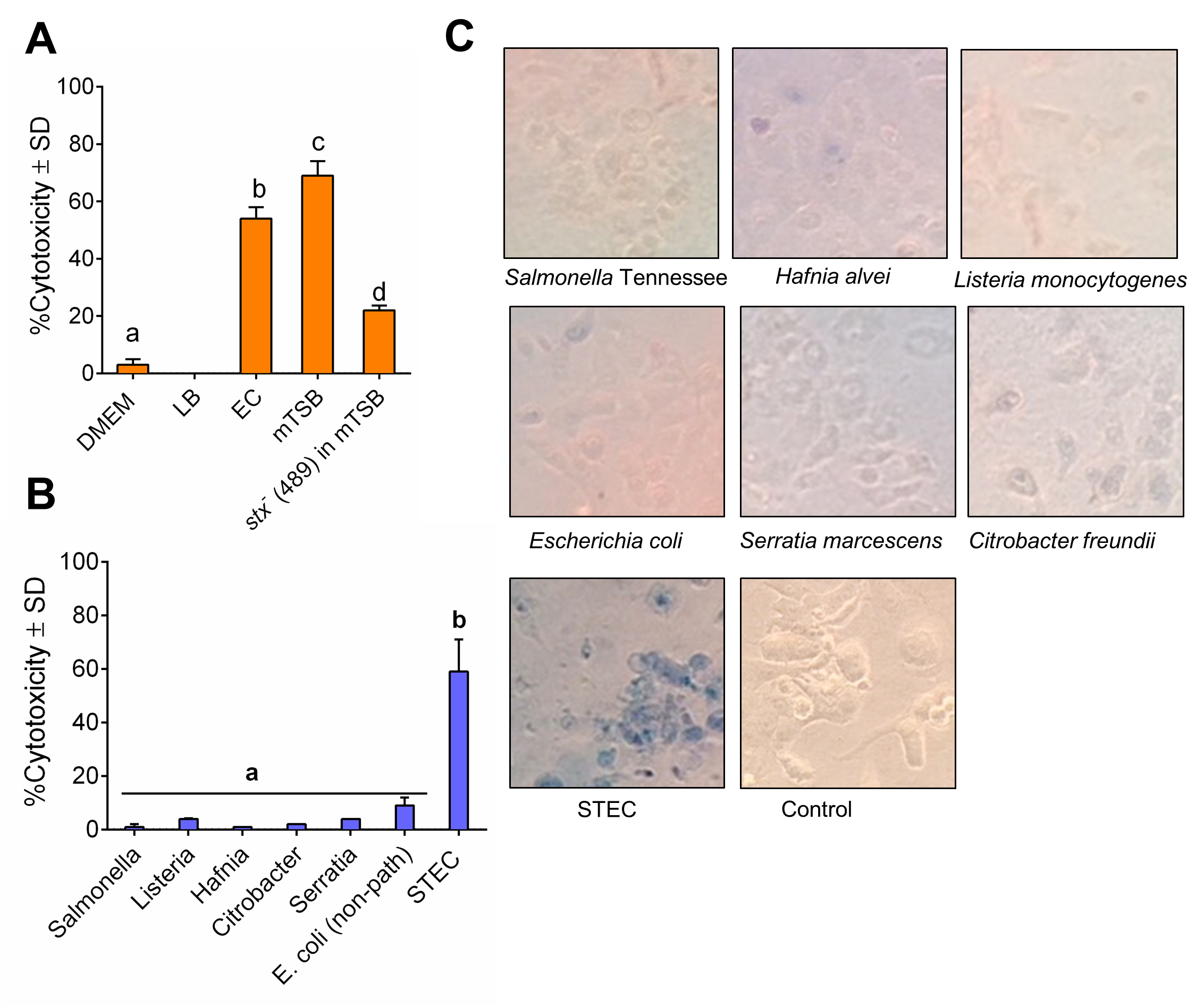

Supplement: Figure S1 — Specificity assay. (A) Effect of growth media (DMEM, LB, EC, mTSB, stx-) on Vero cytotoxicity. (B,C) Cytotoxicity of non-STEC bacteria on 3D Vero cells after 6 h exposure. (C) STEC strain EDL933 caused severe cell damage, cell rounding, and cell death visualized after Trypan blue staining while other pathogens (Salmonella Tennessee, Listeria monocytogenes, Serratia marcescens, Citrobacter freundii, Hafnia alvei, and Escherichia coli) did not cause any damage during this period (magnification, 400×). [file Image_1.TIF]
